# Compensatory T-type Ca^2+^ channel activity alters D2-autoreceptor responses of *Substantia nigra* dopamine neurons from Cav1.3 L-type Ca^2+^ channel KO mice

**DOI:** 10.1038/srep13688

**Published:** 2015-09-18

**Authors:** Christina Poetschke, Elena Dragicevic, Johanna Duda, Julia Benkert, Antonios Dougalis, Roberta DeZio, Terrance P. Snutch, Joerg Striessnig, Birgit Liss

**Affiliations:** 1 Institute of Applied Physiology, University of Ulm, 89081 Ulm, Germany; 2 Djavad Mowafaghian Centre for Brain and Health and Michael Smith Laboratories, University of British Columbia, V6T1Z4 Vancouver, Canada; 3 Institute of Pharmacy, Department of Pharmacology and Toxicology, University of Innsbruck, 6020 Innsbruck, Austria

## Abstract

The preferential degeneration of Substantia nigra dopamine midbrain neurons (SN DA) causes the motor-symptoms of Parkinson’s disease (PD). Voltage-gated L-type calcium channels (LTCCs), especially the Cav1.3-subtype, generate an activity-related oscillatory Ca^2+^ burden in SN DA neurons, contributing to their degeneration and PD. While LTCC-blockers are already in clinical trials as PD-therapy, age-dependent functional roles of Cav1.3 LTCCs in SN DA neurons remain unclear. Thus, we analysed juvenile and adult Cav1.3-deficient mice with electrophysiological and molecular techniques. To unmask compensatory effects, we compared Cav1.3 KO mice with pharmacological LTCC-inhibition. LTCC-function was not necessary for SN DA pacemaker-activity at either age, but rather contributed to their pacemaker-precision. Moreover, juvenile Cav1.3 KO but not WT mice displayed adult wildtype-like, sensitised inhibitory dopamine-D2-autoreceptor (D2-AR) responses that depended upon both, interaction of the neuronal calcium sensor NCS-1 with D2-ARs, and on voltage-gated T-type calcium channel (TTCC) activity. This functional KO-phenotype was accompanied by cell-specific up-regulation of NCS-1 and Cav3.1-TTCC mRNA. Furthermore, in wildtype we identified an age-dependent switch of TTCC-function from contributing to SN DA pacemaker-precision in juveniles to pacemaker-frequency in adults. This novel interplay of Cav1.3 L-type and Cav3.1 T-type channels, and their modulation of SN DA activity-pattern and D2-AR-sensitisation, provide new insights into flexible age- and calcium-dependent activity-control of SN DA neurons and its pharmacological modulation.

Dopaminergic (DA) neurons within the *Substantia nigra* (SN) are of particular interest, as their selective loss causes the major motor related symptoms of Parkinson’s disease (PD)^1^,^2^,^3^. The underlying cause for the high susceptibility of SN DA neurons to PD-triggers is still not clear^4^. However, activity-dependent calcium signalling and associated mitochondrial stress, selectively in SN DA neurons contribute to their high vulnerability to degeneration^4^. More precisely, L-type voltage-gated Ca^2+^ channels (LTCCs) are active during spontaneous, intrinsically generated pacemaker-activity in SN DA neurons, resulting in dendritic Ca^2+^ oscillations, and also related oscillations of mitochondrial membrane potentials and oxidative stress levels^5^. Of clinical relevance, epidemiological studies indicate that systemic administration of blood-brain-barrier permeable LTCC-blockers of the dihydropyridine (DHP) type (e.g. isradipine) reduce the risk for developing PD by about 30%^6^,^7^,^8^. Further, in PD animal-models, DHPs seem to protect SN DA neurons from degeneration in both non-human primates^9^ and mice — in a dose-dependent manner^10^.

Both pore-forming subunits of brain LTCCs, Cav1.2 and Cav1.3, are expressed in SN DA neurons^11^,^12^, but biophysical and pharmacological findings^13^,^14^ point to a unique contribution of Cav1.3 LTCCs to PD^6^,^15^. The physiological functions of Cav1.3 LTCCs in SN DA neurons are, however, still largely unclear^16^, and an age-dependent role of LTCCs for SN DA pacemaker-activity^17^ is highly disputed^17^,^18^,^19^. Recently, we identified a novel role of Cav1.3 LTCCs in SN DA neurons for an age-dependent modulation of somatodendritic dopamine D2-autoreceptor (D2-AR) responses^11^. D2-ARs reduce SN DA activity through activation of G-protein-coupled, inwardly-rectifying potassium channels (GIRK2)^20^,^21^. In juvenile mice, Cav1.3 LTCCs can adapt SN DA activity in response to high extracellular dopamine-levels (elevated *in vivo* e.g. by L-DOPA or cocaine) by providing the Ca^2+^ source for neuronal calcium sensor NCS-1^22^ dependent sensitisation of inhibitory D2-AR responses^11^.

Here we analysed Cav1.3 KO mice, using cell-specific electrophysiological and molecular brain-slice techniques in order to elucidate the roles of Cav1.3 LTCCs in SN DA neurons. We compared Cav1.3 KO findings with effects of acute pharmacological inhibition of LTCCs as well as of voltage-gated T-type Ca^2+^ channels (TTCCs)^23^. As age-dependent postnatal differences in SN DA pacemaker-control are described^11^,^17^, we analysed both, juvenile (PN13) and adult (PN90) mice. While LTCC function was not crucial for SN DA pacemaker-frequency at either age, TTCCs in contrast modulated SN DA pacemaker-activity in an age-dependent manner. Furthermore, juvenile Cav1.3 KO mice displayed adult-like sensitised SN DA D2-AR responses that depended on internal calcium and NCS-1/D2-AR interaction, as well as on TTCC activity, and that were accompanied by the selective up-regulation of Cav3.1 TTCC and of NCS-1 mRNA.

## Results

### Age-dependent changes of afterhyperpolarisation (AHP) in SN DA neurons from Cav1.3 KO mice point to a compensatory KO phenotype

**Figure 1:**
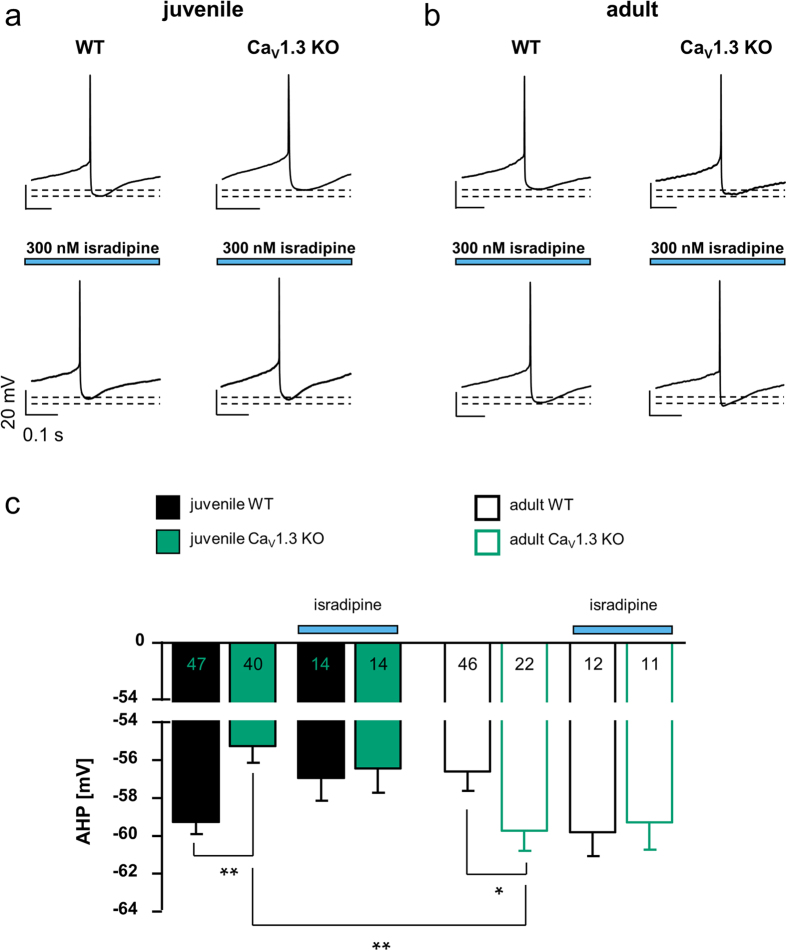
Age-dependent changes of afterhyperpolarisation (AHP) in Cav1.3 KO mice. (**a**/**b)** Whole-cell current clamp recordings of single action potentials of SN DA neurons from juvenile (**a**) and adult (**b**) WT and Cav1.3 KO mice, under control conditions and in the presence of L-type Ca^2+^ channel blocker (300 nM isradipine, blue bars). Dashed lines indicate −60 mV and −55 mV, respectively; scale bars: 20 mV/0.1 s. (**c**) Bar graphs display mean values ± SEM, and number of neurons analysed (n-values). WT data are given in black, Cav1.3 KO data in green. Note that SN DA AHPs from juvenile KO mice are significantly smaller while those of adult KO are significantly larger than those of respective WT mice. Significant differences are marked by asterisks. Data values and statistics are detailed in Table 1.

We first extensively compared a variety of basal biophysical properties of SN DA neurons from juvenile (PN13) and adult (PN90) wildtype (WT) and Cav1.3 KO mice using whole-cell patch-clamp analysis of *in vitro* brain-slices. No striking biophysical differences were observed (data not shown). However, the Ca^2+^-dependent afterhyperpolarisation (AHP) of spontaneous action potentials of SN DA neurons showed small but significant changes in Cav1.3 KO mice (Fig. 1 and Table 1). While the AHP was significantly smaller in SN DA neurons from juvenile Cav1.3 KO compared to those of juvenile WT (juvenile WT: −59.27 mV ± 0.6, n = 47; juvenile Cav1.3 KO: −55.26 mV ± 0.9, n = 40; U-value of the Wilcoxon-Mann-Whitney-U test, WMWU = 560, p = 0.001), it was significantly larger in SN DA neurons from adult Cav1.3 KO mice compared to those of adult WT (adult WT: −56.52 mV ± 0.8, n = 46; adult Cav1.3 KO: −59.75 mV ± 1.1, n = 22; WMWU = 327, p = 0.02). Of further note to these AHP changes is that the acute block of both, Cav1.2 and Cav1.3 LTCCs, by 300 nM isradipine had no effect on the AHPs of SN DA neurons from WT and Cav1.3 KO mice. The concentration of 300 nM isradipine was carefully chosen to avoid unspecific off-target effects (as described e.g. in^17^), while still providing efficient full block of Cav1.2 and Cav1.3 LTCCs^6^,^11^,^24^ (see methods for details). These findings point to an age-dependent compensatory SN DA phenotype in the Cav1.3 KO mouse.

### Acute pharmacological LTCC-block or chronic Cav1.3 loss does not affect pacemaker frequency of SN DA neurons

**Figure 2:**
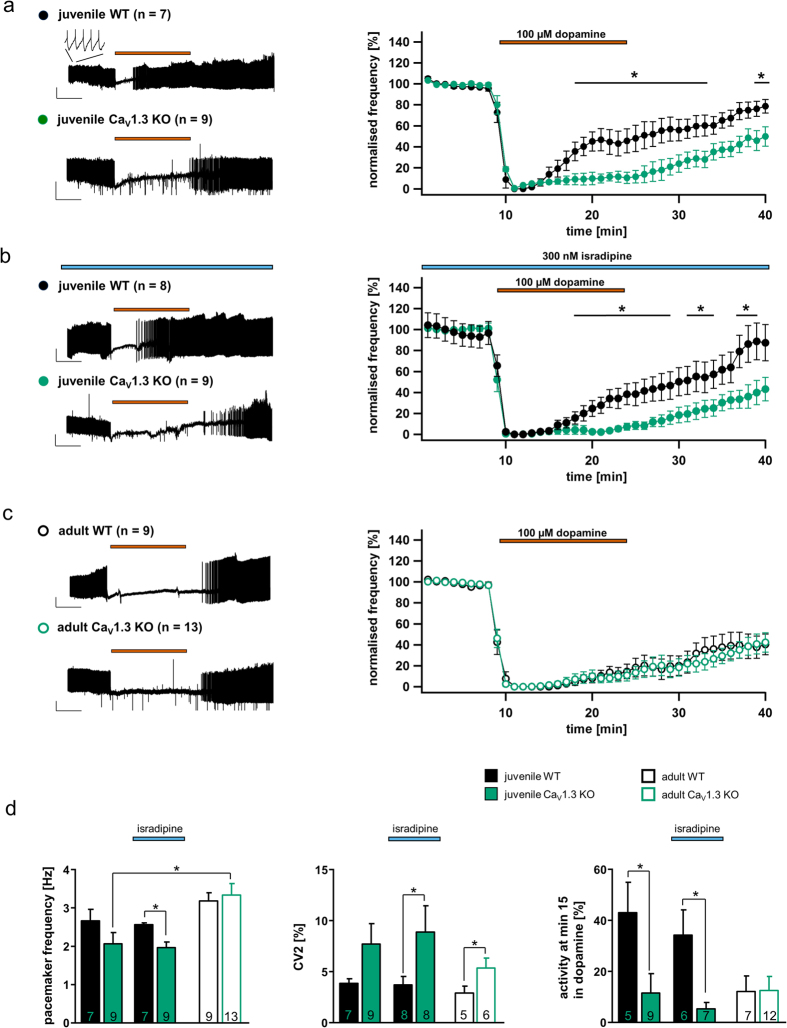
Adult-like, sensitised D2-autoreceptor responses in SN DA neurons from juvenile Cav1.3 KO mice. (**a**–**c**) **Left:** Perforated-patch clamp recordings of spontaneous activity of SN DA neurons from juvenile (**a**/**b**) and adult (**c**) WT and Cav1.3 KO mice. Application of dopamine (100 μM) is indicated by red bars, (pre-)incubation of isradipine (300 nM) is indicated by blue bars. Scale bars 20 mV/5 min. **Right:** Normalised frequencies plotted against time for all analysed SN DA neurons. Note the absence of a desensitisation of dopamine D2-autoreceptor (D2-AR) responses in juvenile KO mice compared to WT, and that in contrast, acute block of L-type Ca^2+^ channels (LTCCs) did not alter D2-AR responses of juvenile WT or KO mice. (**d**) **Left:** Mean SN DA pacemaker frequencies before dopamine application. Note that neither chronic loss of Cav1.3 nor acute LTCC-block (isradipine) altered SN DA pacemaker frequency. **Middle:** Mean SN DA pacemaker precision, given as the less firing-rate dependent interspike interval derived CV2 values. **Right:** SN DA D2-AR responses, given as mean relative spontaneous activity at the last minute of dopamine application (min 15) in relation to respective basal pacemaker frequencies. Note that the prominent desensitisation of D2-AR responses of juvenile SN DA neurons from WT mice was absent in the KO, but not in acute LTCC-blocker. All data are shown as the mean ± SEM. WT data are shown in black and KO data in green. Significant differences are marked by asterisks. Data values and statistics detailed in Table 1.

A possible age-dependent contribution of LTCCs to generation of pacemaker-activity of SN DA neurons is controversial^17^,^18^,^19^. Thus, we analysed the spontaneous pacemaker frequency and its precision (given as the coefficient of variation (CV) of the interspike interval (ISI), and as CV2 values) of SN DA neurons from juvenile and adult WT and Cav1.3 KO mice using *in vitro* brain-slices. Further, we analysed pacemaker-activity in response to acute dopamine application (100 μM) in order to address D2-AR responses. Perforated patch-clamp recordings allowed unperturbed physiological activity and signalling of SN DA neurons to be assessed. As summarised in Fig. 2d (left) and Table 1, pacemaker frequencies of juvenile or adult SN DA neurons were neither significantly altered due to acute LTCC pharmacological inhibition, nor affected by chronic Cav1.3 loss in the global KO mice. However, LTCCs seem to stabilise pacemaker precision, since Cav1.3 KO neurons displayed less regular pacemaker-activities as evident from the respective CV2 values (compare Fig. 2d and Table 1; CV2-values: juvenile WT: 3.87% ± 0.4, n = 7; juvenile Cav1.3 KO: 7.71% ± 2, n = 9; WMWU = 19, p = 0.2; juvenile WT isradipine: 3.73% ± 0.8, n = 8; juvenile Cav1.3 KO isradipine: 8.88% ± 2.6, n = 8; WMWU = 12, p = 0.04; adult WT: 2.92% ± 0.7, n = 5; adult Cav1.3 KO: 5.35% ± 1, n = 6; WMWU = 4, p = 0.05).

### SN DA neurons from juvenile Cav1.3 KO mice exhibit sensitised, adult-like dopamine D2-autoreceptor responses

In contrast to basal pacemaker-activity, the response to dopamine was dramatically different in SN DA neurons from juvenile Cav1.3 KO mice compared to those of WT. As summarised in Fig. 2a–d right and Table 1, and as recently described^11^, inhibitory dopamine D2-AR responses of SN DA neurons from juvenile WT mice display a prominent desensitisation that is not observed in adult WT SN DA neurons. Contrastingly, SN DA neurons from juvenile Cav1.3 KO mice displayed sensitised D2-AR responses, resembling those of adult WT and Cav1.3 KO mice (Fig. 2a,c,d). Given our previous finding that acute pharmacological Cav1.3 block can prevent D2-AR sensitisation of juvenile WT SN DA neurons in response to *in vivo* elevated extracelullar dopamine levels^11^, these Cav1.3 KO findings were somewhat unexpected, as we had rather expected the opposite result: namely prominently desensitising D2-ARs in SN DA neurons of juvenile Cav1.3 KO mice. To further examine this discrepancy, and given that our AHP-findings pointed to a compensatory phenotype in the Cav1.3 KO mouse (see Fig. 1), we analysed the effect of acute LTCC-block (300 nM isradipine) on SN DA D2-AR responses of juvenile WT and Cav1.3 KO mice.

As shown in Fig. 2b,d (right), isradipine had neither an effect on desensitising D2-AR responses of juvenile WT mice, nor on sensitised D2-AR responses of juvenile Cav1.3 KO mice. As we had previously shown that 300 nM isradipine is sufficient to fully block sensitised Cav1.3-dependent D2-AR responses in juvenile WT SN DA neurons^11^, these findings strongly suggest that a compensatory mechanism rather than the general lack of Cav1.3 is responsible for the altered D2-AR responses observed in the juvenile Cav1.3 KO mice. These pharmacological data also make it highly unlikely that functional compensation is mediated by Cav1.2 LTCCs, as isradipine blocks both, Cav1.3 and Cav1.2 channels — with Cav1.2 being more sensitive to isradipine^25^, and it did not affect the D2-AR responses of SN DA neurons from juvenile Cav1.3 KO mice (Fig. 2b,d).

### Sensitised D2-autoreceptor responses in SN DA neurons from juvenile Cav1.3 KO mice depend on free internal calcium and on neuronal calcium sensor NCS-1/D2-AR interaction

**Figure 3:**
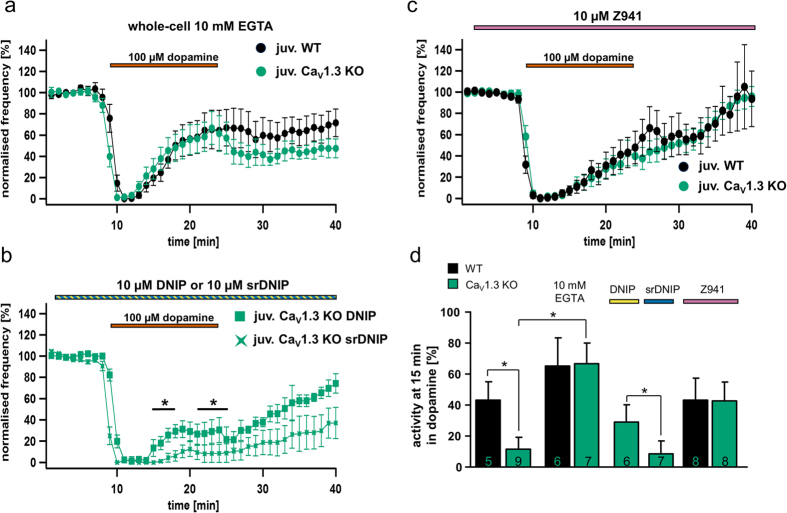
Calcium-dependent, sensitised D2-autoreceptor responses in SN DA neurons from juvenile Cav1.3 KO mice are mediated by T-type Ca^2+^ channels and the neuronal calcium sensor NCS-1. Dopamine D2-AR experiments and SN DA data presentation are similar to that for Fig. 2. (**a)** Experiments in the presence of 10 mM EGTA (whole-cell current clamp), to buffer free internal Ca^2+^ in SN DA neurons from juvenile WT (n = 6) and Cav1.3 KO (n = 7) mice. (**b**) Experiments in the presence of either DNIP (yellow, D2/NCS-1 interacting peptide) or scrambled DNIP (as controls, blue, srDNIP) to block D2-AR/NCS-1 interactions in SN DA neurons from juvenile Cav1.3 KO mice (DNIP: n = 11; srDNIP: n = 10, perforated patch or on-cell recordings). Mean basal pacemaker frequencies were not affected by DNIP (see Table 1). (**c**) Experiments in the presence of the T-type Ca^2+^ channel blocker Z941 (10 μM; purple bar, perforated patch) in SN DA neurons from juvenile WT (n = 10) and Cav1.3 KO (n = 8). (**d**) Activity of SN DA neurons from (**a**–**c**) (and from Fig. 2a) at the last minute of dopamine application (min 15). Note that buffering of free internal Ca^2+^ (EGTA) is inducing prominent D2-AR desensitisation in both SN DA neurons from juvenile WT and KO mice, while DNIP (but not srDNIP), as well as Z941, both introduce WT-like D2-AR desensitisation in SN DA neurons from juvenile Cav1.3 KO mice. All data are shown as mean ± SEM, WT data in black and KO data in green. Significant differences are marked by asterisks. Data values and statistics are detailed in Table 1.

To further define a functional compensatory phenotype in juvenile Cav1.3 KO mice, we next studied whether the mechanism of SN DA D2-AR desensitisation downstream of Cav1.3 was similar in the Cav1.3 KO to the Ca^2+^ sensor NCS-1 mechanism that we identified for WT mice^11^. To determine if D2-AR desensitisation in SN DA neurons from juvenile Cav1.3 KO mice depended on free intracellular Ca^2+^ and interaction of NCS-1 with D2-ARs, we buffered internal Ca^2+^ with 10 mM EGTA (whole-cell; Fig. 3a,d and Table 1), and in a second experiment we applied a membrane permeable peptide that prevents D2-R/NCS-1 interactions (perforated patch; DNIP, or scrambled DNIP (srDNIP) as control^11^,^26^; Fig. 3b,d and Table 1). Internal Ca^2+^ buffering induced prominently desensitising D2-AR responses in SN DA neurons from both, juvenile WT and Cav1.3 KO mice (Fig. 3b,d and Table 1). Furthermore, the presence of the DNIP peptide (but not srDNIP) re-stored WT-like desensitising D2-AR responses in SN DA neurons from juvenile Cav1.3 KO mice (Fig. 3b,d and Table 1). These findings strongly suggest that the D2-AR desensitisation mechanism downstream of the Ca^2+^ source is not altered in SN DA neurons from Cav1.3 KO mice and relies — as in WT — on Ca^2+^-dependent NCS-1/D2-AR interactions^11^. These findings also indicate an alternative, compensatory Ca^2+^ source in SN DA neurons from Cav1.3 KO mice, mediating NCS-1/D2-AR interactions and the observed reduction in D2-AR desensitisation.

### Sensitised D2-autoreceptor responses in SN DA neurons from juvenile Cav1.3 KO mice depend on voltage-gated T-type calcium channel activity

To address a compensatory Ca^2+^ source in SN DA neurons from Cav1.3 KO mice, we tested the effect of the specific T-type Ca^2+^ channel (TTCC) blocker Z941^27^. We probed for TTCC effects as they have been shown to provide the major Ca^2+^ source for the AHP in juvenile mouse SN DA neurons, which is mediated by Ca^2+^ -activated small-conductance K^+^ (SK) channels^28^,^29^,^30^, and because we had detected changes in AHPs in the Cav1.3 KO mouse (Fig. 1). As summarised in Fig. 3c,d and Table 1, 10 μM of Z941 restored WT-like, desensitising D2-AR responses in SN DA neurons from juvenile KO mice, while those of WT mice were not affected. The TTCC-blocker Z944 (10 µM) had a similar effect (data not shown). These findings strongly suggest a compensatory up-regulation of T-type Ca^2+^ channel currents, and/or functional coupling of TTCCs with D2-ARs via NCS-1 in SN DA neurons of juvenile Cav1.3 KO mice. Z941 had however no effect on the sensitised D2-AR responses of adult SN DA neurons, neither from WT nor from Cav1.3 KO mice (see Supplementary Figure 1, and Table 1).

### Age-dependent T-type Ca^2+^ channel modulation of pacemaker activity in SN DA neurons

**Figure 4:**
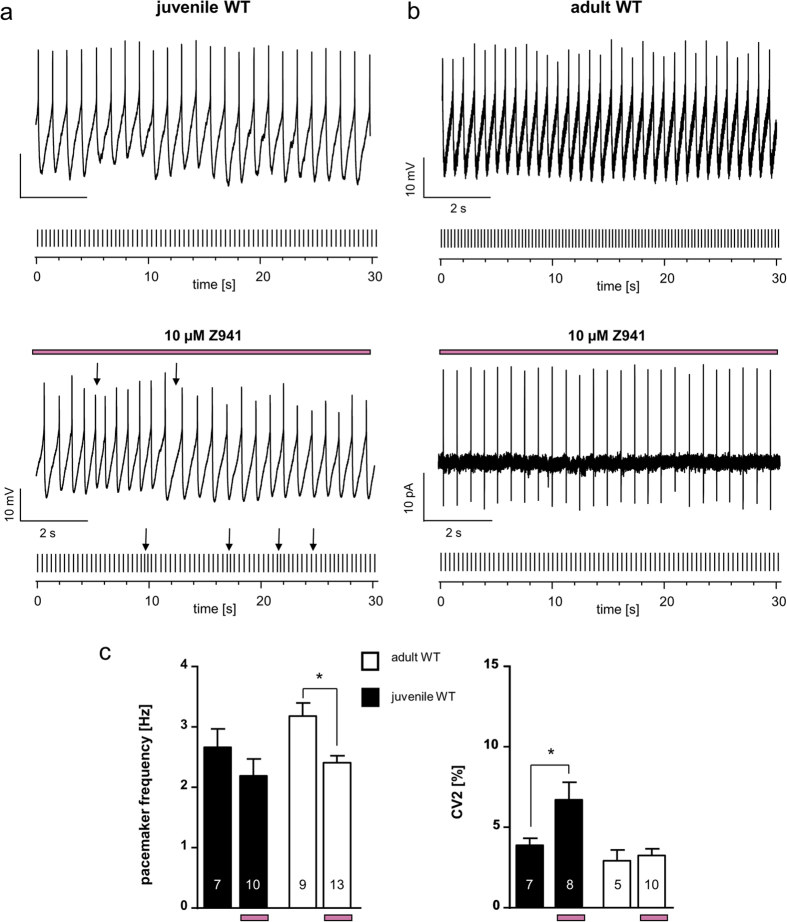
Age-dependent T type Ca^2+^ channel modulation of pacemaker activity in SN DA neurons. (**a**/**b**) Exemplary perforated patch-clamp or cell-attached recordings of spontaneous activity of SN DA neurons from juvenile (**a**) and adult (**b**) WT mice without and with T-type channel inhibitor Z941 (pre-)incubation (10 μM, indicated by purple bars). Under each original 10 second trace, a 30 seconds schematic spike train representation is given. Arrows point to irregularity in pacemaker-activity in SN DA neurons from juvenile WT mice in Z941. (**c**) Data analysis as in Fig. 2 (**d**) **Left:** Mean SN DA pacemaker frequencies. Note that Z941 significantly reduced SN DA pacemaker frequency of adult WT SN DA but not of juvenile WT SN DA neuron. **Right:** Mean SN DA pacemaker precision, given as CV2 values. Note that Z941 significantly reduced pacemaker precision of juvenile WT SN DA but not adult WT SN DA neurons. All data are shown as mean ± SEM. Significant differences are marked by asterisks. Data values are detailed in Table 1.

Given the functional coupling of D2-AR sensitisation to T-type Ca^2+^ channels, selectively in SN DA neurons from juvenile Cav1.3 KO mice, we addressed physiological age-dependent functional roles of TTCCs in wild-type SN DA neurons by comparing pacemaker activity and its precision in juvenile and adult mice. As summarised in Fig. 4 and Table 1, we gained evidence for differential functions of T-type currents in SN DA neurons from juvenile and adult WT mice. In juveniles, TTCC-activity stabilised pacemaker precision (CV2: juvenile WT: 3.87% ± 0.4, n = 7; juvenile WT in Z941: 6.69% ± 1; WMWU = 9, p = 0.03), similar as previously described^28^,^30^, but did not change pacemaker activity. Of further note, the AHP differences between WT and Cav1.3 KO were abolished by TTCC-block (juvenile WT Z941: −59.06 mV ± 0.8, n = 17; juvenile Cav1.3 KO Z941: −58.4 mV ± 1.6, n = 14; WMWU = 118, p = 1, see Table 1). In adults in contrast, TTCC-block did not affect pacemaker precision, but reduced SN DA pacemaker frequency by about 30% (pacemaker frequency: adult WT: 3.2 Hz ± 0.2, n = 9; adult WT in Z941: 2.4 Hz ± 0.1, n = 13; WMWU = 20, p = 0.009). Furthermore, this impact of Z941 on adult SN DA pacemaker frequency was significantly pronounced in Cav1.3 KO compared to those of adult WT (pacemaker frequency: adult Cav1.3 KO: 3.3 Hz ± 0.3, n = 13; adult Cav1.3 KO in Z941: 1.4 Hz ± 0.1; WMWU = 3, p < 0.0001; adult WT in Z941 vs. adult Cav1.3 KO in Z941: WMWU = 6, p < 0.0001, see Table 1). This finding, together with the altered AHPs in SN DA neurons from Cav1.3 KOs (Fig. 1), point to an upregulation of TTCC currents in Cav1.3 KO.

### SN DA neurons from juvenile Cav1.3 KO mice exhibit larger amplitudes of fast-inactivating low-voltage-activated, T-type calcium channel currents

**Figure 5:**
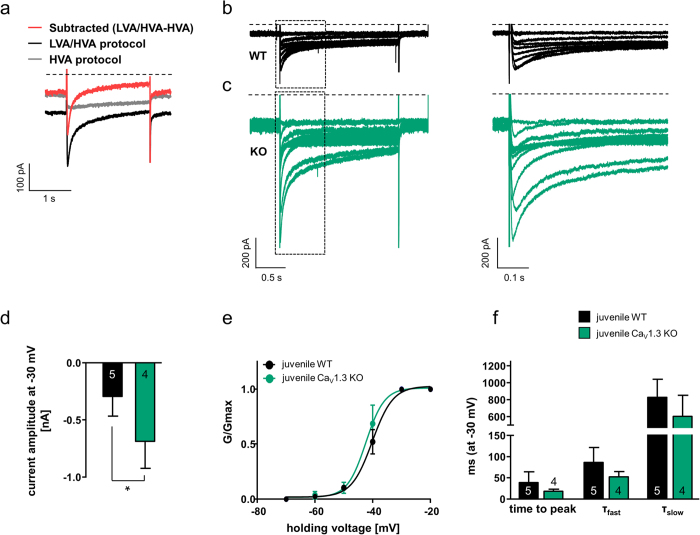
Larger amplitudes of fast-inactivating low-voltage activated, T-type calcium channel currents in SN DA neurons from juvenile Cav1.3 KO mice. (**a**) Overlay of representative currents recorded from a juvenile WT SN DA neuron in response to a single step to −30 mV from a LVA/HVA composite protocol (holding current of −100 mV, black trace) and from a HVA protocol (holding current of −60 mV, gray trace), to discriminate between low and high voltage activated currents (LVA/HVA). The red trace represents the subtracted current. Currents were evaluated for their time to peak. The subtracted LVA currents and the LVA/HVA composite current were consistently fitted with a two exponential decay (tau fast [τ_fast_] and tau slow [τ_slow_]). Scale bar 100 pA/1s. (**b/c**) Representative traces of subtracted, fast-inactivating T-type calcium channel blocker Z944-sensitive barium currents in juvenile SN DA neurons from a WT and a Cav1.3 KO mouse (response to 10 mV incremental depolarising pulses to 0 mV from a holding potential of −100 mV). Dotted boxes indicates the expanded view of the left hand traces shown on the right. Currents exhibited a voltage-dependent fast-activation and voltage-dependent fast-inactivation. Scale bars: left traces: 200 pA/1 s; right traces: 200 pA/500ms. (**d**) Maximal current amplitude of T-type barium currents in juvenile WT and Cav1.3 KO SN DA neurons at a test voltage of −30 mV. Note that the peak amplitude is significantly (about 3-fold) larger in Cav1.3 KO, suggesting elevated T-type currents (WMWU = 0, p = 0.02). (**e**) Steady-state activation curves for putative T-type barium currents in WT and Cav1.3 KO mice. Plot represents the ratio of conductance (G) to the maximal conductance (Gmax) and has been fitted with a single Boltzmann equation to identify the voltage for half-maximal activation (V50) and the slope (s) of the steady-state activation curve. Note similar steady-state activation of T-type currents in WT and KO. (**f**) Kinetic properties (time to peak and inactivation time constants τ_fast_ and τ_slow_) of T-type currents at a test voltage of −30 mV are similar in SN DA neurons from juvenile WT and Cav1.3 KO mice. All data are shown as mean ± SEM, WT data in black and KO data in green. Significant differences are marked by asterisks.

To compare TTCC currents in SN DA neurons from juvenile WT and Cav1.3 KO mice, we performed whole-cell voltage-clamp recordings. To isolate fast-inactivating, T-type Ca^2+^ currents we exploited current subtraction between two voltage-clamp protocols used to discriminate between low- and high-voltage-activated currents (HVA and LVA), together with specific intracellular/extracellular solutions that block sodium, potassium and synaptic conductances, as described previously^31^. To reduce the amount of inactivation seen when isolating Ca^2+^ currents in SN DA neurons under voltage-clamp protocols (e.g.^32^ and for easier discrimination between fast-inactivating and persistent currents), we used barium ions instead of calcium ions as the charge carrier^33^. Neurons were held for 5 seconds at either −100 mV (LVA/HVA composite current protocol) or −60 mV (HVA protocol), and were depolarised in 10 mV increments (for 2 s, every 6 s) up to +20 mV to construct full current-voltage (I–V) curves. Current subtraction was used to compute the peak amplitude of the fast-inactivating LVA currents recruited with the LVA/HVA composite protocol by subtracting the slowly-activating currents recorded with the HVA protocol (Fig. 5). Subtraction of currents resulted in isolation of fast activating, fast-inactivating conductances in SN DA neurons from WT mice (Fig. 5a). The subtracted current exhibited voltage-dependence in its activation, becoming faster at more positive voltages (time to peak at −50 mV: 62.1 ms ± 16.2, n = 5; at 0 mV, 26.0 ms ± 6.4, n = 5, p = 0.049, paired t-test, data not shown). The inactivation phase was consistently better fitted with the sum of two exponentials (τ_fast_ and τ_slow_) that contributed on average 50–70% and 30–50% of the total current amplitudes. The fast inactivation time constant exhibited some voltage-dependency in most cells becoming faster throughout the −50 to 0 mV voltage range, the slow inactivation constant did not exhibit changes in the range studied (τ_fast_ and τ_slow_ at −50 mV: 152.1 ms ± 46.4 and 646.6 ms ± 162.5 respectively; at 0 mV: 75.1 ms ± 6.4 and 676.0 ms ± 67.6 respectively; τ_fast_ p = 0.2, τ_slow_ p = 0.9 with paired t-tests for inter-voltage τ_fast_ and τ_slow_ comparisons, n = 5, data not shown). Since we pharmacologically occluded fast-activating sodium and potassium conductances, and since biophysical properties of the fast-inactivating subtracted current are very similar to the T-type calcium currents recorded previously in SN DA neurons in brain slices^32^, and are pharmacologically inhibited by 10 µM Z944 (n = 14, data not shown), we refer to them henceforth as T-type (barium) currents. Comparing maximal T-type (barium) current amplitudes obtained at −30 mV from juvenile SN DA neurons from WT (n = 5 neurons from 4 mice, representative traces in Fig. 5b) and Cav1.3 KO mice (n = 4 neurons from 3 mice, representative traces in Fig. 5c) revealed that Cav1.3 KO mice displayed significantly larger peak currents (compare Fig. 5d; current amplitude at −30 mV: juvenile WT: −294.4 p ± 77.1, n = 5; juvenile Cav1.3 KO: −686.1 pA ± 118.4, n = 4; WMWU = 0, p = 0.02). SN DA T-type currents from juvenile WT and Cav1.3 KO mice however exhibited similar parameters for the steady-state activation (WT and Cav1.3 KO: voltage for half-maximal activation (V_50_): −40.1 mV ± 0.8, n = 5 and −42.3 mV ± 1.3, n = 4, WMWU = 6, p = 0.4; slope (s): 3.6 ± 1.0, n = 5 and 3.2 ± 1.2, n = 4, WMWU = 5, p = 0.3, see Fig. 5e) and activation/inactivation kinetics (WT and Cav1.3 KO at −30 mV: time to peak: 39.1 ms ± 11.3 and 18.6 ms ± 2.3, WMWU = 3, p = 0.1; τ_fast_: 86.3 ms ± 15.9 and 52.5 ms ± 6.2, WMWU = 2, p = 0.06; τ_slow_: 827.2 ms ± 95.9 and 604.9 ms ± 123.8, WMWU = 4, p = 0.2; n = 5 and 4 respectively, Fig. 5f). These data suggest that the steady state biophysical and kinetic properties of SN DA T-type (barium) currents are not per se affected in the Cav1.3 KO mice, but the peak current amplitude is about 3-fold larger. This would be in line with an elevated expression of TTCCs in SN DA neurons from juvenile Cav1.3 KO mice.

### SN DA neurons from Cav1.3 KO mice exhibit elevated mRNA levels selectively for the T-type Ca^2+^ channel subunit Cav3.1 as well as for NCS-1

**Figure 6:**
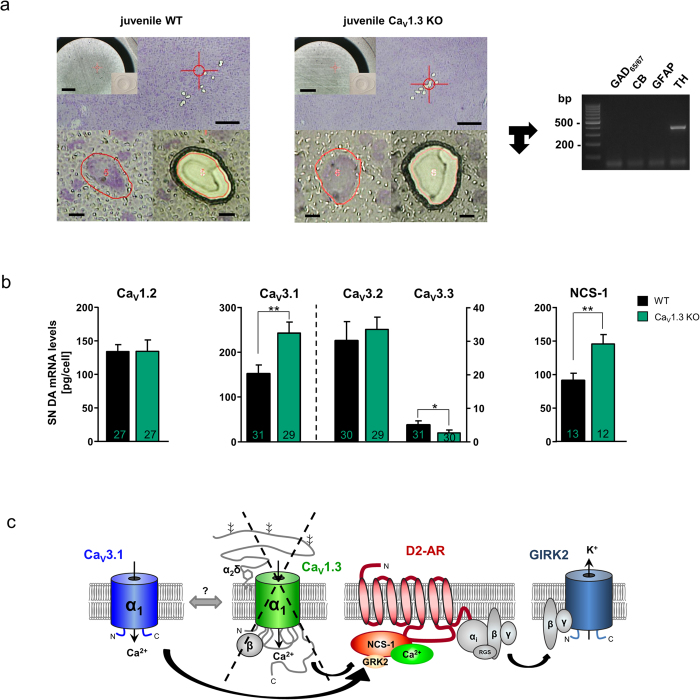
Compensatory up-regulation of Cav3.1 T-type Ca^2+^ channel subunits and of NCS-1 mRNA in SN DA neurons from juvenile Cav1.3 KO mice. (**a**) Overview of WT (left) and Cav1.3 KO mouse (middle) coronal midbrain sections after UV-laser-microdissection (UV-LMD) of 10 individual SN DA neurons each. Scale bars: 250 μm. **Inserts:** photograph of the reaction-tube-cap for inspection of collection of all 10 neurons after UV-LMD, prior to cell lysis and reverse transcription. Scale bars: 500 μm. **Lower left/middle:** individual SN DA neurons before and after UV-LMD. Scale bars: 10 μm. **Right:** Multiplex nested PCR results (2% agarose-gel-electrophoresis). All analysed SN DA cDNA pools were PCR-positive for tyrosine hydroxylase (TH) and negative for calbindind_28k_ (CB), for GABAergic markers (GAD65/67) and for astroglial marker (GFAP). (**b)** Cell-specific quantitative RT-PCR data of the L-type Ca^2+^ channel subunit Cav1.2, of the T-type Ca^2+^ channel subunits Cav3.1, Cav3.2, and Cav3.3, and of the neuronal calcium sensor NCS-1, for SN DA neurons from juvenile WT and Cav1.3 KO mice (n numbers given in bars). Data given as [pg/cell] in respect to a cDNA standard curve, generated from WT mouse midbrain tissue. All data given as the mean ± SEM. WT data given in black, KO data in green. Note significantly higher mRNA-levels of Cav3.1 and NCS-1 in SN DA from Cav1.3 KO. (**c**) Cartoon summarising the postulated molecular mechanism of altered dopamine NCS-1/D2-AR/GIRK2 channel signalling in SN DA neurons from juvenile Cav1.3 KO mice: Chronic loss of Cav1.3 L-type-Ca^2+^ -channels (with its regulatory β- and α2δ subunits) that sensitises D2-ARs in response to extracellular dopamine via the neuronal calcium sensor NCS-1 is functionally compensated in SN DA neurons by Cav3.1 T-type-Ca^2+^ -channels, boosting calcium-dependent D2-AR/NCS-1 interaction, that prevent GRK2-mediated D2-AR phosphorylation and thus β-arrestin-mediated receptor internalisation and desensitisation—resulting in enhanced GIRK2- mediated SN DA pacemaker-activity inhibition. Abbreviations: D2-AR: D2-autoreceptor, GIRK2: G-protein-coupled, inwardly rectifying K^+^ channel 2, GRK2: G-protein coupled kinase 2, RGS: regulator of G-protein signalling; for details see text.

To address a possible upregulated expression of TTCC subunits, and to molecularly define the underlying nature of the compensatory functional coupling of TTCCs with D2-ARs via NCS-1, we quantified mRNA levels of all three TTCC subtypes (Cav3.1, Cav3.2 and Cav3.3 α1-subunits), as well as of Cav1.2 and NCS-1 in juvenile SN DA neurons from WT and Cav1.3 KO mice, by combining UV-LMD and RT-PCR approaches (Fig. 6). All three TTCC subunits were expressed in mouse SN DA neurons, although Cav3.1 was the by far most abundant TTCC subunit, while Cav3.2 mRNA-levels were about ~8-fold lower, and Cav3.3 mRNA was not detected in most analysed SN DA neurons (Fig. 6b). More importantly, mRNA-levels of only the Cav3.1 subtype were significantly increased (by ~50%) in SN DA neurons of juvenile Cav1.3 KO mice compared to those of WT (WT Cav3.1: 159.4 ± 19.8 pg/cell, n = 31; Cav1.3 KO Cav3.1: 241.2 ± 21.4 pg/cell, n = 29; WMWU = 267, p = 0.006; Fig. 6b). In contrast, mRNA levels of Cav3.2 and Cav3.3 were not elevated (WT Cav3.2: 30.06 ± 5.7 pg/cell, n = 30; Cav1.3 KO Cav3.2: 33.28 ± 3.7 pg/cell, n = 29; WMWU = 351, p = 0.2; WT Cav3.3: 4.9 ± 1.1 pg/cell, n = 31, with only 18 cell-pools giving a PCR positive signal; Cav1.3 KO Cav3.3: 2.5 ± 0.8 pg/cell, n = 30 with only 9 cell-pools giving a PCR positive signal; WMWU = 331.5, p = 0.03). Similarly, mRNA-levels of Cav1.2 were not changed (WT Cav1.2: 134.1 ± 10.2 pg/cell, n = 27; Cav1.3 KO Cav1.2: 134.6 ± 17 pg/cell, n = 27; WMWU = 347, p = 0.8.). These data identify Cav3.1 as the prominent TTCC channel in WT SN DA neurons and its mRNA upregulation in SN DA neurons from Cav1.3 KO mice, likely mediating the compensatory functionally coupled TTCC/D2-AR phenotype, accompanied by significantly elevated fast-inactivating low-voltage-activated TTCC mediated currents in SN DA neurons from Cav1.3 KO mice.

An increased functional expression of Cav3.1 channels and respective TTCC currents alone might be sufficient to explain the observed Ca^2+^ and NCS-1 dependent D2-AR sensitisation in SN DA neurons from Cav1.3 KO mice, especially as TTCCs have a significantly lower activation threshold compared to Cav1.3 LTCCs^34^,^35^,^36^. However, additionally elevated levels of NCS-1, the molecular linker between voltage-gated Ca^2+^ channels and Ca^2+^ dependent D2-AR desensitisation, could further augment the observed functional Cav1.3 KO phenotype. As evident (Fig. 6), NCS-1 mRNA levels were also significantly increased (by ~50%) in SN DA neurons from juvenile Cav1.3 KO mice compared to those of WT (WT NCS-1: 91.2 pg/cell ± 10.5, n = 13; Cav1.3 KO NCS-1: 145.0 pg/cell ± 13.2, n = 12; WMWU = 29, p = 0.007). For data stratification, and to minimise effects of possible sampling artefacts, we normalised Cav3.1 and NCS-1 RT-qPCR data to respective SN DA cell-sizes, and determined similar significantly higher mRNA levels selectively for Cav3.1 and NCS-1 (Cav3.1 WT vs. Cav1.3 KO: WMWU = 256, p = 0.01; Cav3.2 WT vs. Cav1.3 KO: WMWU = 318, p = 0.23; Cav3.3 WT vs. Cav.13 KO: WMWU = 333, p = 0.08; NCS-1 WT vs. Cav1.3 KO: WMWU = 24, p = 0.002).

We conclude that elevated functional expression of NCS-1 and Cav3.1 T-type channels—and their functional coupling to D2-AR signalling—underlies the TTCC-blocker sensitive D2-AR sensitisation in SN DA neurons from Cav1.3 KO mice (Fig. 6c).

## Discussion

In the present report we utilise cell-specific electrophysiological and molecular biological analysis of brain-slices to examine the functional phenotype of juvenile (PN13) and adult (PN90) SN DA neurons from a general Cav1.3 KO mouse compared to wildtype mice. Our findings further clarify the physiological functions of Cav1.3 LTCCs in SN DA neurons. This is of particular relevance as Cav1.3 LTCC activity in SN DA neurons has been assumed to render these neurons particularly vulnerable to PD-triggers and to neurodegeneration^5^,^6^,^7^,^8^,^9^, LTCC blockers are already in clinical trials as neuroprotective PD-therapy^37^,^38^, and novel Cav1.3 selective LTCC-blockers are in preclinical development^15^.

In summary, our findings strongly suggest that Cav1.3 is not crucial for SN DA pacemaker-activity at either postnatal age, however we identified a novel age-dependent role of T-type Ca^2+^ channels for SN DA pacemaker-modulation. (Cav1.3) LTCCs rather stabilise pacemaker-precision and modulate inhibitory dopamine-autoreceptor (D2-AR) sensitisation via the neuronal calcium sensor NCS-1. D2-ARs control activity of SN DA neurons in a negative feedback loop, but they are also involved in PD-pathology and are targets in pharmacological PD-therapy^11^,^21^,^39^. Of particular importance, we detected a functional Cav3.1 T-type Ca^2+^ channel compensation in SN DA neurons from Cav1.3 L-type Ca^2+^ channel KO mice, providing a novel homeostatic link between those channels, with implications for the intended chronic pharmacological block of Cav1.3 channels as PD-therapy, where this flexible signalling network might also need to be considered.

The role of LTCCs, in particular of the Cav1.3 subtype with its more negative activation threshold compared to Cav1.2^13^, for the generation and/or modulation of pacemaker-activity in SN DA neurons is controversial. A postnatal age-dependent switch from HCN (hyperpolarisation-activated cyclic nucleotide-gated) cation-channel driven pacemaker in juvenile mouse SN DA neurons to a significantly more metabolically challenging Cav1.3 LTCC driven pacemaker in adult SN DA neurons has been suggested^17^, but is highly disputed^11^,^18^,^19^,^40^,^41^,^42^,^43^. LTCCs seem to stabilise rather than generate pacemaker-activity in SN DA neurons^19^,^44^. Our results, obtained from Cav1.3 KO and WT mice (CV2 values, Table 1 and Fig. 2d) support the view that LTCC-function is not crucial for the generation of pacemaker-activity in either juvenile or adult SN DA neurons, but contributes to the stabilisation of pacemaker precision. These findings are in agreement with a recent study, describing a less precise pacemaker in SN DA neurons from older (25–30 month) mice, presumably accompanied by reduced LTCC currents^40^. A less precise pacemaker in SN DA neurons from Cav1.3 KO mice would be also in line with a contribution of LTCCs to modulating Ca^2+^ — and SK-channel dependent AHPs of SN DA neurons (Fig. 1), as the AHP in mouse juvenile SN DA neurons is crucial for the precision of their pacemaker-activity^30^. Interestingly, in SN DA neurons from adult PARK-gene PD-model mice (Pink1 and HtrA2/Omi KO) impaired intracellular Ca^2+^ signalling had also been described, that lead to a functional reduction of SK channel activation, accompanied by a more irregular pacemaker and a higher tendency for burst activity^32^ — further linking altered Ca^2+^ homeostasis and impaired related ion channel function in SN DA neurons to their altered activity, high vulnerability to degeneration, and to PD. While LTCCs might also contribute to SK-mediated AHPs in juvenile WT SN DA neurons^41^,^45^,^46^, they seem to be predominantly functionally coupled to T-type Ca^2+^ channels^28^,^30^. In line with this role of TTCCs in juvenile WT SN DA neurons, TTCC-block with Z941 lead to less precise pacemaker-activities (increased CV2 values, Table 1) in juvenile WT SN DA neurons (Fig. 4). In juvenile SN DA neurons from Cav1.3, the AHP was not pronounced but reduced (Fig. 1), TTCC-block abolished these differences, and pacemaker-activity was less precise, compared to WT, independent from Z941 block. Together, these findings would argue for a functional uncoupling of T-type currents from SK channels in SN DA neurons from Cav1.3 KO mice, and thus pacemaker precision in juvenile KO — likely due to compensatory T-type channel upregulation, accompanied by altered spatio-temporal calcium-levels, as described^47^,^48^,^49^. In contrast, in adult WT SN DA neurons, block of T-type currents did not affect pacemaker precision but reduced pacemaker frequency by about 30%. This age-dependent switch of T-type channel contribution to pacemaker activity in SN DA neurons *in vitro* has to our knowledge not been reported before. Furthermore, in adult Cav1.3 KOs the AHP was pronounced (Fig. 1), and the impact of TTCC-inhibition on SN DA pacemaker frequency of adult KOs was significantly stronger (about 50% reduced activity) compared to adult WT (Fig. 4 and Table 1) — again in line with a functional upregulation of T-type currents in the Cav1.3 KO. Our whole-cell voltage-clamp data further support that the maximal T-type channel mediated (barium) current amplitude is significantly ~3-fold larger in SN DA neurons from juvenile Cav1.3 KO mice, while steady state biophysical and kinetic properties of T-type currents were similar (see Fig. 5). These findings are in line with elevated expression of TTCCs and a compensatory functional upregulation of TTCCs in SN DA neurons of Cav1.3 KO mice. Through quantifying dopamine D2-AR responses of WT and KO SN DA neurons, we identified indeed a functional TTCC compensation of the chronic Cav1.3 LTCC loss in SN DA neurons (reduction in D2-AR desensitisation in Cav1.3 is disrupted if NCS-1/D2-AR interactions are prevented by the DNIP-peptide, and it is also abolished in the presence of the T-type channel blockers Z941 and Z944 (data for Z944 not shown)). This functional TTCC-phenotype is likely mediated by the most abundant Cav3.1 TTCC isoform that is upregulated at the mRNA level in SN DA neurons from Cav1.3 KO mice.

Cav1.3 LTCCs activate faster and at more negative potentials compared to Cav1.2 (and all other high-voltage-activating (HVA) Cav1.x/Cav2.x voltage-gated Ca^2+^ channels^13^,^50^). Accordingly, we found no functional or molecular evidence for an enhanced compensatory Cav1.2 LTCC function in SN DA neurons from Cav1.3 KO mice (Figs 2 and 6), in accordance with earlier reports for whole brain and sinoatrial node^24^,^25^. Cav3.1 channels in contrast to Cav1.2 activate at even more negative membrane potentials than Cav1.3^34^,^35^,^36^, and their functional compensation in Cav1.3 KO mice suggests that an inward Ca^2+^ current at (sub-)threshold membrane potentials is likely to be crucial for proper physiological SN DA neuron function. In addition to TTCC currents and Cav3.1 mRNA, we also detected elevated mRNA levels of the neuronal Ca^2+^ sensor NCS-1 in SN DA neurons from juvenile Cav1.3 KO mice (Fig. 6). As NCS-1 prevents Ca^2+^ -dependent D2-receptor internalisation and receptor-desensitisation^51^, we conclude that the observed sensitised, Ca^2+^-and NCS-1-dependent D2-AR responses in SN DA neurons from juvenile Cav1.3 KO mice point to a flexible, homeostatic Ca^2+^-and dopamine-dependent inhibitory NCS-1/D2-AR signalling pathway^39^,^43^, that likely is crucial for related physiological function of SN DA neurons^22^.

How might the expression of Cav3.1 TTCCs and of NCS-1 be selectively up-regulated in SN DA neurons in Cav1.3 KO mice ? Ca^2+^ channel subtypes exhibit distinct spatial, temporal and disease-related expression-patterns^36^,^50^, and the neuronal transcriptional control of Cav3.1 is selectively regulated^52^. LTCCs on the other hand mediate calcium-dependent gene-transcription via CREB-dependent signalling^53^. Thus, a direct link between transcriptional Cav3.1 up-regulation and the loss of Cav1.3 LTCC activity is not unreasonable. Strikingly, it has recently been reported, that the C-terminus of Cav1.3 can translocate to the nucleus and directly alter gene-expression, of e.g. SK2 channels^54^. In line, a direct calcium-dependent transcriptional stimulation of NCS-1 is also reported^55^ that could explain the elevated NCS-1 mRNA levels.

Lower threshold voltage-gated Cav1.3 LTCCs are active during pacemaking, selectively in SN DA neurons — but not in neighbouring, more resistant VTA DA neurons^56^ — and they also substantially contribute to the oscillatory Ca^2+^ influx within the interspike interval between action potentials of SN DA neurons^41^,^57^. The function of LTCCs appears not to be crucial for SN DA pacemaker-activity, and it thus is likely that their role in affecting oscillatory Ca^2+^ levels represents distinct and essential physiological functions. Intracellular Ca^2+^ covers a wide area of functions in a cell, for instance it can directly activate enzymes such as those of the citrate cycle and the mitochondrial nitric oxide synthase^43^,^58^, and alters gene expression in SN DA neurons via Ca^2+^-dependent gene transcription^59^,^60^. LTCC functions (especially the associated oscillatory intracellular Ca^2+^ burden) however also render SN DA neurons highly vulnerable to PD-triggers and neurodegeneration^5^, particularly as Ca^2+^ is only weakly buffered in SN DA neurons by Ca^2+^ -binding proteins like calbindin_d28k_^6^,^45^. Ca^2+^-and NCS-1-dependent sensitisation of inhibitory D2-AR responses and thus enhanced GIRK2 channel mediated hyperpolarisation and reduction of metabolically demanding activity, may therefore serve an important mechanism to counteract this high metabolic burden and excitotoxicity in SN DA neurons (compare Fig. 6c and^43^,^61^). This mechanism seems to work well under physiological conditions in SN DA neurons from WT as well as Cav1.3 KO mice. However, when PD-trigger factors (such as mitochondrial toxins or familial PD PARK-gene mutations) further challenge SN DA neurons, this metabolic control mechanism might no longer be sufficient^32^,^43^,^61^. In this view, adult SN DA neurons from PARK7 (DJ-1) KO mice indeed display juvenile WT-like, desensitising D2-AR responses^62^, and WT DJ-1 has a similar neuroprotective effect on mouse SN DA neurons as DHP LTCC-blockers (like isradipine) have^5^.

Beyond this scenario, our findings might have implications for the clinical use of LTCC-blockers as neuroprotective PD-therapy. Similar to long-lasting loss of Cav1.3 Ca^2+^ signalling in Cav1.3 KO mice, chronic pharmacological inhibition of Cav1.3 channels by isradipine or novel Cav1.3-selective drugs^15^ may lead to a similar compensatory functional up-regulation of Cav3.1 TTCCs (and/or NCS-1) in SN DA neurons. Whether this might happen or not: based on our findings, Ca^2+^ and NCS-1 mediated D2-AR sensitisation will reduce SN DA activity, and thus could counteract excitotoxicity and its detrimental consequences^43^. Consequently, stimulation of NCS-1/D2-AR signalling in SN DA neurons could be beneficial for their survival. In this view, elevated NCS-1 mRNA levels have been detected in remaining human SN DA neurons from *post mortem* PD patients^11^. Notably, an age-related decline of neuronal Cav3.1 expression has also been described in mice and humans, and it is exacerbated in Alzheimer’s disease^63^. Consequently, further studies are necessary to address the complex cell-specific dynamics and the (patho-) physiological consequences of the here identified novel flexible TTCC-Cav1.3/NCS-1 signalling network in SN DA neurons. In conclusion, our finding that different types of voltage-gated Ca^2+^ channels can compensate each other, modulate SN DA activity-patterns, and serve as alternative Ca^2+^ sources for the same downstream signalling pathway (NCS-1/D2-AR/GIRK2) that is inhibiting the electrical activity of SN DA neurons in a dopamine-dependent fashion, provides new insights into flexible age- and calcium-dependent activity-control of SN DA neurons, that might need to be considered for LTCC-based drug therapy of PD and beyond.

## Materials and Methods

### Animals

Only male mice (~PN13 and ~PN90) were analysed in this study. Mice were bred in the animal facility of Ulm University. Cav1.3 KO mice^64^ were of mixed C57BL/6 × sv129 background, back-crossed three times into C57BL/6. As wildtype (WT) control strains, Cav1.3^+/+^ and C57BL/6 mice were analysed. As both control groups displayed no significant differences data were pooled. All animal procedures/experiments were approved by the Regierungspräsidium Tübingen (Aktenzeichen: 35/9185.81-3.TV-No1043, Reg. Nr. 0.147) and carried out in accordance with the approved guidelines.

### Brain slice preparation and electrophysiological recordings

Preparation of vital coronal brain slices form PN13 and PN90 mice, as well as electrophysiological recordings and pharmacological experiments, were performed essentially as described^11^. For all details, see S1. Briefly, *for pharmacology*, slices were preincubated with 300 nM isradipine to block LTCCs^6^, or 10 μM Z941 or Z944, respectively, to block TTCCs^27^, or with 10 μM DNIP/srDNIP, a D2/NCS-1 interaction prevention peptide^26^, synthesised by Genscript (http://www.genscript.com/index.html), bath applied (in ACSF) for at least 30 minutes prior to recordings, and throughout recordings. The concentration of 300 nM isradipine was carefully chosen as it causes a complete block of L-type currents in sinoatrial node cells, in which the majority of Ca^2+^ current is mediated by Cav1.3 LTCCs^24^. Complete inhibition at this concentration was achieved despite the somewhat lower sensitivity of Cav1.3 as compared to Cav1.2^24^. Furthermore, modelling data predicted that at −50 to −60 mV ~90% of Cav1.3 channels should be antagonised by 100 nM isradipine^6^. In addition, in a recent publication using 300 nM, we could fully block indirect LTCC effects on SN DA D2-AR responses in juvenile SN DA neurons from brain slice preparations in WT as well as in Cav1.2DHP^−/−^ mice^11^. Moreover, we recently reported an IC_50_ for isradipine below 100 nM in a preliminary analysis of recombinant Cav1.3 channels during stimulation with SN DA like electrical activity^65^. *Whole-cell voltage-clamp isolation of T-type barium currents:* Whole-cell, voltage-clamp recordings were performed with an internal solution containing (in mM): 140 tetraethylammonium chloride (TEA-Cl), 5 cesium chloride (CsCl), 10 HEPES, 10 EGTA, 2 MgCl_2_ (pH 7.3, osmolarity 275–285 mosmol/l). The extracellular ACSF solution contained (in mM): 140 TEA-Cl, 5 CsCl, 2 MgCl_2_, 1 BaCl_2_, 1.25 NaH_2_PO_4_, 26 NaHCO_3_, 15 glucose, 2 4-aminopyridine (4-AP), 0.02 gabazine, 0.01 DNQX, 0.001 tetrodotoxin (TTX) and 0.003 isradipine. Series resistance (R_s_) and input resistance (R_in_) were frequently monitored throughout the experiments via at 10 mV, 5 ms depolarising step. Whole-cell capacitive transients were evaluated through a 10 mV step (−50 to −60 mV) and were negated using the amplifier’s circuitry. Currents were low-pass filtered at 3 kHz, collected at 20 kHz using Patchmaster software. Current subtraction was used to compute the peak amplitude of the fast-inactivating LVA currents recruited with the LVA/HVA composite protocol by subtracting the slowly-activating currents recorded with the HVA protocol. Subtracted, LVA currents, were subjected to leak subtraction using a scaled 10 mV depolarising pulse (2 s from −100 mV to −90 mV) that represents the linear background leak current. The amplitude of the subtracted LVA current was measured from its peak to the current plateau 50 ms before the end of the 2 s depolarising test pulse at each test voltage. Channel conductance (G) for any given potential (V) was calculated assuming a reversal potential (Erev) of +100 mV for barium ions using the standard driving force equation, G = I/(V-Erev). Normalised conductance plots (G/Gmax) were fitted with a standard single Boltzmann function (using GraphPad Prism 6) in order to compute the voltage for half-maximal activation (V50) and the slope (s) of the steady-state activation curve for the subtracted fast-inactivating currents. Subtracted LVA currents were also subjected to kinetic analysis by determining the time to peak amplitude (measured from the beginning of the voltage step to the peak of the current) and by fitting their inactivation with two exponential inactivation time constants (fitted from the peak of the current until the current plateau 50 ms before the end of the 2 s depolarising test pulse at each test voltage).

### UV-laser-microdissection and RT-PCR analysis

UV-laser-microdissection (UV-LMD) of SN DA neurons from mouse brain sections, using an LMD7000 system (Leica Microsystems), as well as cell lysis, cDNA synthesis, purification, multiplex nested PCR (for marker-gene analysis) and quantitative real-time PCR (qPCR) of UV-LMD samples was performed essentially as described^11^, for all details and further references, please see S1, and S2 Supplementary Table A/B (providing all primer/assay information).

### Data analysis and statistics

Data analysis was performed using FitMaster (HEKA Elektronik), Neuroexplorer 4 (Plexon Inc.) and Igor Pro 6 (Wavemetrics Inc.), GraphPad Prism 6 (GraphPad Software) or the R project for statistical computing^66^ with RExcel. The coefficient of variation of the interspike intervall (CV ISI) was calculated in Neuroexplorer 4 (analysis: interspike interval histograms; result: Coeff.Var.ISI*100). To minimise influences of different mean firing rates or slow changes, CV2 values were also calculated, by dividing the standard deviation of two adjacent ISIs by their mean, and multiplied with the square root of 2 as described in^67^, (1) with i as the counter.


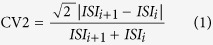


All data are given as mean ± SEM (standard error of the mean). Numbers of electrophysiologically analysed neurons as well as molecularly analysed neuronal pools are given as n-value (from at least 4 different animals each). Data were tested for outliers via the ROUT function of GraphPad Prism 6. Tests for statistically significant differences are specified with the respective results. In general, statistically significant differences between WT and Cav1.3 KO (with/without drugs) were identified via Wilcoxon-Mann-Whitney-U two-sample rank-sum test (WMW) with a defined level of significance of 0.05. For comparisons beyond direct comparisons of WT vs. Cav1.3 KO, either the non-parametric one-way Kruskal-Wallis test and the post-hoc Dunn’s Test for multiple comparisons (DTMC), or a 1-way ANOVA followed by Fishers least significant difference test (FLSD), with an alpha of 0.05 were used. P-values ≤ 0.05 are noted with (*) and ≤ 0.005 with (**) respectively in graphs; the U-value of the WMW test, a measure for test-statistic (in GraphPad “Mann-Whitney U”) is provided as WMWU value.

## Additional Information

**How to cite this article**: Poetschke, C. *et al.* Compensatory T-type Ca^2+^ channel activity alters D2-autoreceptor responses of Substantia nigra dopamine neurons from Cav1.3 L-type Ca^2+^ channel KO mice. *Sci. Rep.* **5**, 13688; doi: 10.1038/srep13688 (2015).

## Supplementary Material

Supplementary Information

## Figures and Tables

**Table 1:** AHP, pacemaker frequency and its precision, as well as D2-AR responses in SN DA neurons from WT and Cav1.3 KO mice.

	control conditions						control conditions					
	juvenile WT	n	juvenile Ca_V_1.3 KO	n	U	p	adult WT	n	adult Ca_V_1.3 KO	n	U	p
afterhyperpolarisation [mV] (whole-cell)	−59.27 ± 0.6	47	−55.25 ± 0.9	40	560	**0.001**	−56.52 ± 0.8	46	−59.75 ± 1.1	22	327	**0.02**
pacemaker frequency [Hz] (five minutes, perforated patch)	2.67 ± 0.3	7	2.07 ± 0.3	9	15.5	0.1	3.18 ± 0.2	9	3.34 ± 0.3	13	55	0.8
CV ISI [%] (perforated patch)	7.71 ± 0.9	7	16.77 ± 4	9	15	0.09	7.13 ± 2	5	11.36 ± 2.3	6	6	0.1
CV2 [%] (perforated patch)	3.87 ± 0.4	7	7.71 ± 2	9	19	0.2	2.92 ± 0.7	5	5.35 ± 1	6	4	**0.05**
activity at min. 15 of dopamine [%] (perforated patch)	43.11 ± 11.9	5	11.55 ± 7.6	9	39	**0.02**	12.13 ± 6.1	7	12.59 ± 5.4	12	49	1
	**in 300 nM isradipine**						**in 300 nM isradipine**					
	juvenile WT	n	juvenile Ca_V_1.3 KO	n	U	p	*adult WT	n	adult Ca_V_1.3 KO	n	U	p
afterhyperpolarisation [mV] (whole-cell)	−56.94 ± 1.2	14	−56.44 ± 1.3	14	88	0.7	−59.81 ± 1.3	12	−59.3 ± 1.5	11	53	0.4
pacemaker frequency [Hz] (five minutes, perforated patch)	2.57 ± 0.04	7	1.96 ± 0.1	9	8	**0.01**	3.19 ± 0.3	9				
CV ISI [%] (perforated patch)	8.85 ± 1.5	8	15.53 ± 3.5	8	17	0.1	9.9 ± 1.2	8				
CV2 [%] (perforated patch)	3.73 ± 0.8	8	8.88 ± 2.6	8	12	**0.04**	5.7 ± 0.7	8				
activity at min. 15 of dopamine [%] (perforated patch)	34.29 ± 9.9	7	5.39 ± 2.5	9	9	**0.01**	1.5 ± 3.2	13				
	**in 10 μM Z941**						**in 10 μM Z941**					
	juvenile WT	n	juvenile Ca_V_1.3 KO	n	U	p	adult WT	n	adult Ca_V_1.3 KO	n	U	p
afterhyperpolarisation [mV] (whole-cell)	−59.06 ± 0.8	17	−58.4 ± 1.6	14	118	1						
pacemaker frequency [Hz] (five minutes, perforated patch)	2.19 ± 0.3	10	2.41 ± 0.3	8	31	0.5	2.4 ± 0.1	13	1.4 ± 0.1	10	6	**<0.0001**
CV ISI [%] (perforated patch)	12.44 ± 1.3	7	12.25 ± 1.4	7	20	0.6	7.05 ± 1.1	11	11.8 ± 1.2	9	12	**0.003**
CV2 [%] (perforated patch)	6.7 ± 1.1	8	6.8 ± 1.2	7	27	0.9	3.3 ± 0.4	10	7.1 ± 1.2	9	10	**0.003**
activity at min. 15 of dopamine [%] (perforated patch)	43.11 ± 14.3	8	42.6 ± 12.1	8	28	0.7	6.32 ± 3.2	13	13.66 ± 9.3	10	97	0.9
							**perforated patch/cell-attached in**					
	**10 mM EGTA (high EGTA, whole-cell)**						**10 μM DNIP**		**10 μM srDNIP**			
	juvenile WT	n	juvenile Ca_V_1.3 KO	n	U	p	juvenile Ca_V_1.3 KO	n	juvenile Ca_V_1.3 KO	n	U	p
afterhyperpolarisation [mV] (whole-cell)	−56.25 ± 0.7	15	−56.99 ± 0.8	19	116	0.4						
pacemaker frequency [Hz] (five minutes)	1.97 ± 0.2	6	2.1 ± 0.2	7	18	0.7	2.6 ± 0.3	11	2.49 ± 0.4	10	50	0.7
CV ISI [%]	11.21 ± 1.1	6	11.31 ± 2	7	17	0.6	12.51 ± 1.7	8	9.12 ± 1.6	4	10	0.3
CV2 [%]	7.05 ± 1	6	6.27 ± 0.9	7	16	0.5	8.06 ± 1.7	8	7.13 ± 1.9	4	14	0.8
activity at min. 15 of dopamine [%]	65.2 ± 18.1	6	66.63 ± 13.5	7	26	0.6	28.95 ± 11.1	6	8.43 ± 8.4	7	7	**0.03**

Presented are mean values ± SEM for afterhyperpolarisation (AHP), basal pacemaker frequency, pacemaker precision (given as coefficient of variation of the interspike interval, CV ISI, as well as CV2 values), and the relative activity at the last minute (min 15) in dopamine in respect to basal pacemaker frequency for SN DA neurons from juvenile and adult WT and Cav1.3 KO mice, under the distinct recording conditions, as indicated. min. = minute, U = U-value from Wilcoxon-Mann-Whitney-U two-sample rank-sum test (WMWU), p = p-value from WMW-test. Additional significant differences according to Kruskal-Wallis and post hoc Dunn’s Test for multiple comparisons (DTMC): AHP: juvenile WT vs. juvenile WT high EGTA: DTMC p = 0.04; juvenile Cav1.3 KO vs. adult Cav1.3 KO: DTMC p = 0.01; mean frequency: juvenile Cav1.3 KO vs. adult Cav1.3 KO: DTMC p = 0.01; activity at last minute of dopamine application: juvenile Cav1.3 KO vs. juvenile Cav1.3 KO high EGTA: DTMC p = 0.007; pacemaker frequency: adult Cav1.3 KO vs. adult Cav1.3 KO in Z941: DTMC p < 0.0001. Additional significant differences according to 1-way ANOVA and Fishers LSD (FSLD) test: CV2: juvenile WT vs. juvenile WT Z941: FLSD p = 0.01; juvenile WT Z941 vs. adult WT Z941: FLSD p = 0.001; pacemaker frequency: adult WT vs. adult WT Z941: FLSD p = 0.01. *perforated patch data adapted from^11^.
