# Supplementary material for: Compensatory T-type Ca2+ channel activity alters D2-autoreceptor responses of Substantia nigra dopamine neurons from Cav1.3 L-type Ca2+ channel KO mice
Source: Sci Rep. 2015 Sep 18;5:13688. doi: 10.1038/srep13688 (PMC4585382; doi:10.1038/srep13688)
Supplement: Supplementary Information [file srep13688-s1.pdf]

Compensatory T-type Ca^{2+} channel activity alters D2-autoreceptor responses of Substantia nigra dopamine neurons from Cav1.3 L-type Ca^{2+} channel KO mice.

Christina Poetschke, Elena Dragicevic, Johanna Duda, Julia Benkert, Antonios Dougalis, Roberta DeZio, Terrance P. Snutch, Joerg Striessnig, Birgit Liss

Supplementary Information

S1: Supplementary Material and Methods

Electrophysiology

For electrophysiological data acquisition, an EPC10plus patch-clamp amplifier with PulseFit or PatchMaster software (HEKA Elektronik) was used. Data were digitalised with 10 kHz (or for 40 minute perforated patch recordings with 5 kHz to keep file size small), and filtered with Bessel Filter 1: 10 kHz; Bessel Filter 2: 5 kHz. All recordings were performed at a bath temperature of 33°C ± 1. Temperature was controlled using the Badcontroler V (Luigs & Neumann), and checked by a hand thermometer prior and after the recording. All chemicals if not stated otherwise were obtained from Sigma Aldrich. To block fast synaptic transmission, 10µM DNQX disodium salt (Tocris) and 10 µM Gabazine (Tocris) were added to the ACSF. Patch pipettes were pulled from borosilicate glass (GC150TF-15, Harvard Apparatus) using a DMZ Universal Puller (Zeitz-Instrumente GmbH) with program 10 in its default settings but without tip polishing (for details see http://www.zeitz-inst.homepage.t-online.de/Support/Manual/6-Tables/kapitel66-tables.html). For whole-cell current clamp recordings, patch pipettes (2.0-3.0 MΩ,) were filled with internal solution (mM: 135 K-gluconate, 5 KCl, 10 HEPES, 0.1 EGTA (low EGTA) or 10 EGTA (high EGTA), 2 $MgCl_2$, 0.1 NaGTP and 5 MgATP, pH 7.33).

For perforated patch recordings (patch-pipettes: 2.0-2.5 MΩ), the low EGTA internal solution was used without NaGTP and MgATP, but with a final concentration of 100 µg/ml gramicidin. To analyse dopamine autoinhibition of SN DA neurons, a minimum of 5 minutes stable baseline activity either in gramicidine perforated patch or in cell-attached configuration were recorded. This was followed by application of 100 µM dopamine hydrochloride in ACSF via bath-perfusion for 15 minutes and by a 20 minute washout phase. Note that not all analysed neurons allowed a stable full 40 minute recording.

UV-laser-microdissection and RT-PCR analysis

UV-laser-microdissection (UV-LMD) of SN DA neurons from mouse brain sections, using an LMD7000 system (Leica Microsystems), as well as cell lysis, cDNA synthesis, purification, multiplex nested PCR (for marker-gene analysis) and quantitative real-time PCR (qPCR) of UV-LMD samples was performed essentially as described in (Liss, 2002; Grundemann et al., 2011; Dragicevic et al., 2014; Schlaudraff et al., 2014). Details about all used primers are found in Supplemental Tables A and B. cDNA was redissolved after precipitation in 17 µl molecular biology grade water (5PRIME), and stored at -20°C until PCR amplification. Only cDNA samples that were multiplex-PCR positive for tyrosine hydroxylase (TH), and negative for calbindin$_{d28k}$ (CB), l-glutamate decarboxylase (GAD$_{65/67}$) and for glial fibrillaric acidic protein (GFAP) were further analysed by qPCR. This was probed with 5 µl, i.e. 5/17, reflecting ~3 SN DA neurons of each cDNA pool, derived from 10 SN DA neurons. For qPCR assays, TaqMan® probes were used, labelled with 6-carboxyfluorescein (FAM) as reporter and a non-fluorescent quencher (NFQ). Qualitative primers and PCR amplicon details are given in Supplemental Table 1A. Quantitative PCR assay details, and assay-specific standard curve parameters are given in Supplemental Table 1B. Standard curves for quantitative PCR quantification were generated using serial dilutions of cDNA (40, 4, 0.4 and 0.04 ng), derived from mouse midbrain tissue mRNA (RNeasy® MINI kit, Qiagen), as templates in duplicates in at least n = 3 independent qPCR runs. The cDNA amount per cell in relation to the standard was calculated according to:

$$cDNA\ amount\ per\ cell = \frac{S^{\left[\frac{C_t - y_{intercept}}{slope}\right]}}{No_{cells} \cdot cDNA\ fraction}$$

Where: S = serial dilution factor of the standard curve (i.e. 10), No_{cells} = number of harvested neurons per sample (i.e. 10), cDNA fraction = fraction of the UV-laser-microdissection cDNA reaction sample used as template in the individual qPCR reactions (i.e. 5/17 for Cav1.2, Cav1.3, Cav3.1, Cav3.2, NCS-1 and 10/17 for Cav3.3). The $Y_{intercept}$ unit-magnitude corresponds to the respective standard used, (i.e. pg equivalents of standard cDNA). Single cell cDNA amounts were calculated with a $Y_{intercept}$ of 43 for T-type Ca^{2+} channels, 42 for NCS-1 and 45 for L-type Ca^{2+} channels. If samples gave no positive qPCR result, those samples were not omitted from the analysis, but included, by adding 2 CT-values to the highest detected sample (Stahlberg et al., 2013). For further stratification, data were normalised to mean cell-size, derived from each individual respective cell pool, as described (Muhling et al., 2014).

Supplementary References

Dragicevic E, Poetschke C, Duda J, Schlaudraff F, Lammel S, Schiemann J, Fauler M, Hetzel A, Watanabe M, Lujan R, Malenka RC, Striessnig J, Liss B (2014) Cav1.3 channels control D2-autoreceptor responses via NCS-1 in substantia nigra dopamine neurons. Brain.

Grundemann J, Schlaudraff F, Liss B (2011) UV-laser microdissection and mRNA expression analysis of individual neurons from postmortem Parkinson's disease brains. Methods in molecular biology 755:363-374.

Liss B (2002) Improved quantitative real-time RT-PCR for expression profiling of individual cells. Nucleic Acids Res 30:e89.

Muhling T, Duda J, Weishaupt JH, Ludolph AC, Liss B (2014) Elevated mRNA-levels of distinct mitochondrial and plasma membrane Ca(2+) transporters in individual hypoglossal motor neurons of endstage SOD1 transgenic mice. Frontiers in cellular neuroscience 8:353.

Schlaudraff F, Grundemann J, Fauler M, Dragicevic E, Hardy J, Liss B (2014) Orchestrated increase of dopamine and PARK mRNAs but not miR-133b in dopamine neurons in Parkinson's disease. Neurobiol Aging 35:2302-2315.

Stahlberg A, Rusnakova V, Forootan A, Anderova M, Kubista M (2013) RT-qPCR work-flow for single-cell data analysis. Methods 59:80-88.

S2: Supplementary Table A/B: Details of primers and qPCR assays, PCR-amplicons, and qPCR standard curve parameters.

Gene	Primer	Sequence (5`-3`)	Genbank accession no. (NCBI)	5`-Position	Amplicon size [bp]
Calbindin-d28k (CB)	mCB-D28K-F	CGCACTCTCAAACTAGCCG	M21531	87	891
	mCB-D28K-R	CAGCCTACTTCTTTATAGCGCA		977	
	mCB-D28K-F	GAGATCTGGCTTCATTTCGAC		167	440
	mCB-D28K-R	AGTTCCAGCTTTCCGTCATTA		606	
Glial fibrillary acidic protein (GFAP)	mGFAP-F	AGAACAACCTGGCTGCGTAT	K01347	407	786
	mGFAP-R	GCTCCTGCTTCGAGTCCTTA		1192	
	mGFAP-F	AGAAAGGTTGAATCGCTGGA		472	517
	mGFAP-R	CCAGGGCTAGCTTAACGTTG		988	
Glutamate decarboxylase variant 65 (GAD_{65})	mGAD65-F	CATACGCAGACAGCACGTTT	NM_008078.1	166	905
	mGAD65-R	AAAAGATTCCATCGCCAGAG		1070	
	mGAD65-F	GGGATGTCAACTACGCGTTT		606	389
	mGAD-R	CACAAATACAGGGGCGATCT		994	
Glutamate decarboxylase variant 67 (GAD_{67})	mGAD67-F	TGACATCGACTGCCAATACC	Z49976	731	1105
	mGAD67-R	GGGTTAGAGATGACCATCCG		1835	
	mGAD67-F	CATATGAAATTGCACCCGTG		761	702
	mGAD67-R	CGGTGTCATAGGAGACGTCA		1462	
Tyrosine hydroxylase (TH)	mTH-F	CACCTGGAGTACTTTGTGCG	M69200	387	1139
	mTH-R	CCTGTGGGTGGTACCCTATG		1525	
	mTH-F	TGCACACAGTACATCCGTCA		936	377
	mTH-R	TCTGACACGAAGTACACCGG		1312	

Supplementary Table A: Mouse qualitative multiplex PCR (outer) and nested PCR (inner) primers (forward F, reverse R), as well as PCR-amplicon information.

					standard curve data					
**Gene**	**Assay ID**	**Probe Sequence 5‘-FAM (6-carboxyfluorescein), 3‘-NFQ (non-flourescent quencher)**	**Genbank accession no. (NCBI)**	**Amplicon size [bp]**	**exon spanning**	**threshold**	**Y-intercept**	**slope**	**R^2**	**n**
mCav1.2 (Cacna1c)	Mm01188822_m1	TCGAAGGGTGGCCAGAGCTGCTGTA	NM_001159533.1	65	26-27	0.6	44.27 ± 0.32	-3.34 ± 0.08	0.98 ± 0.01	3
mCav1.3 (Cacna1d)	Mm00551392_m1	TCCCTCCAGCTGGTGATGATGAGGT	NM_001083616	62	38-39	0.6	46.19 ± 0.58	-3.22 ± 0.14	0.97 ± 0.02	3
mCav3.1 (Cacna1g)	Mm00486549_m1	GTACCACGAGCAGCCCGAGGAGCTC	NM_009783	57	9-10	0.3	43.01 ± 0.26	-3.33 ± 0.05	0.99 ± 0.00	4
mCav3.2 (Cacna1h)	Mm00445358_m1	ACCGGGTCCCCAGCATGCGAATCCT	NM_021415	52	5-6	0.5	43.41 ± 0.25	-3.19 ± 0.05	0.99 ± 0.00	3
mCav3.3 (Cacna1i)	Mm01299029_m1	AACCGTGTTCCCAGCATGCGGATTC	NM_001044308	60	5-6	0.3	44.27 ± 0.31	-3.43 ± 0.06	0.99 ± 0.00	3
mNCS-1	Mm00490549_m1	CGAGAACAAGGATGGCAGGATTGAG	NM_019681	73	3-4	0.3	41.67 ± 0.45	-3.9 ± 0.11	1.00 ± 0.00	3

Supplementary Table B: Mouse quantitative TaqMan qPCR assay and standard curve informations. FAM - 6-carboxyfluorescein, NFQ - non-fluorescent quencher.

S3: Supplementary Figure

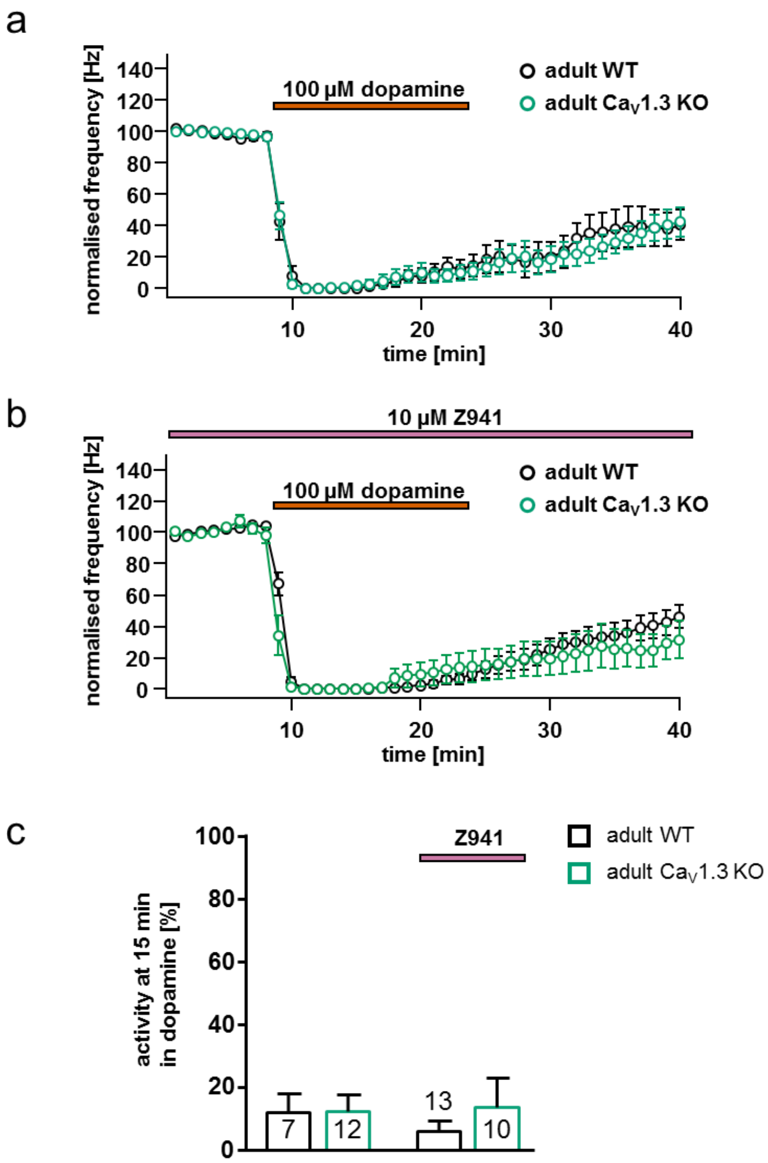

Supplementary Figure: T-type Ca^{2+} channel inhibition does not affect sensitised dopamine D2-AR responses of adult SN DA neurons from WT or Cav1.3 KO mice.

a/b: Perforated-patch clamp / on-cell recordings of spontaneous activity of SN DA neurons from adult WT and Cav1.3 KO mice. Frequencies, normalised against five minutes of baseline frequency (mean values ± SEM), plotted against time for all analysed SN DA neurons. Application of dopamine (100 µM, 15 minutes) is indicated by red bars, (pre-)incubation with T-type channel inhibitor (10 µM Z941) is indicated by purple bars. **c:** SN DA D2-AR responses, given as mean relative spontaneous activity at the last minute of dopamine application (minute 15). Bar graphs display mean values ± SEM, and number of neurons analysed (n-values). Note the sensitised D2-AR responses of adult SN DA neurons from WT and Cav1.3 KO mice, that were not altered due to T-type channel inhibition (in contrast to the Z941-sensitive, sensitised SN DA D2-AR responses from juvenile KO mice, compare Figure 3). WT data in black, KO data in green. Significant differences are marked by asterisks. Data values and statistics detailed in Table 1.
